# *Glycyrrhiza uralensis* Polysaccharide Modulates Characteristic Bacteria and Metabolites, Improving the Immune Function of Healthy Mice

**DOI:** 10.3390/nu17020225

**Published:** 2025-01-09

**Authors:** Wangdi Song, Taifeng Zhang, Yunyun Wang, Shengnan Xue, Yan Zhang, Genlin Zhang

**Affiliations:** 1 State Key Laboratory Incubation Base for Green Processing of Chemical Engineering, School of Chemistry and Chemical Engineering, Shihezi University, Shihezi 832003, China; 2 Key Laboratory of Xinjiang Endemic Phytomedicine Resources Ministry of Education, College of Pharmacy, Shihezi University, Shihezi 832003, China; 3 Testing Center of Xinjiang Tianye Co., Ltd., Shihezi 832099, China

**Keywords:** *Glycyrrhiza* polysaccharide, immunoregulatory activity, gut microbiota, postbiotics, probiotic mechanism

## Abstract

Objectives: Polysaccharides from *Glycyrrhiza* are known to have several bioactive effects. Previous studies have found that low-molecular-weight *Glycyrrhiza* polysaccharide (GP1) is degraded by *Muribaculum_sp_H5* and promotes the production of beneficial bacteria and metabolites, which improves immune disorder and intestinal injury, and then enhances the body’s immune regulation ability. However, the immune regulation effect of GP1 on a healthy body has not been studied. In this study, we aimed to reveal the immune enhancement effect and mechanism of GP1 on healthy mice. Methods: The cytotoxicity and immunomodulatory activity of GP1 were analyzed by cell experiment; the effects of GP1 on antioxidation, immune regulation and gut microbiota structure of healthy body were studied in vivo. In addition, the mechanism of GP1 enhancing immune response of healthy body was analyzed by multi-omics. Results: The results show that GP1 enhanced the immune function of healthy mice by increasing the index of immune organs, improving the organizational structure of immune organs, and increasing the secretion of immune cytokines and immunoglobulin. GP1 also increased the contents of antioxidant factors such as total antioxidant capacity (T-AOC), glutathione peroxidase (GSH-Px) and superoxide dismutase (SOD) in various organs and reduced the content of oxide malondialdehyde (MDA), thus enhancing the body’s antioxidant capacity, promoting cell proliferation and prolonging life. Moreover, GP1 promoted the proliferation of beneficial bacteria, including *Muribaculaceae_unclassified*, *Muribaculum*, *Prevotellaceae_UCG-001*, and *Paramuribaculum*, and the production of characteristic metabolites (collectively referred to as postbiotics), including α-tocopherol, arachidonic acid, melibiose, taurine, and nicotinic acid. These beneficial bacteria and postbiotics have been proven to have health maintaining functions. Conclusions: GP1 promoted the proliferation of beneficial bacteria and increased the production of postbiotics, which should be the mechanism of its beneficial effect. It is expected to be a promising immune dietary supplement.

## 1. Introduction

Prebiotics are components in food that have not been digested by digestive enzymes. Prebiotics can stimulate the proliferation of one or more probiotics, and inhibit the growth or expression of pathogens, which have a positive impact on host health. Prebiotics include sugar polyols, polysaccharides, oligosaccharides, and resistant starch [1,2]. Polysaccharides, including plant, animal, and microbial polysaccharides, are high-molecular-weight carbohydrates formed by glycosidic bonds connecting monosaccharides. Plant polysaccharides are an active component extracted from plants, characterized by a complex structure, high activity, and low toxicity. Plant polysaccharides cannot be degraded by enzymes encoded in the human genome after being ingested into the body but are degraded into monosaccharides or oligosaccharides after reaching the intestine, as the carbon source of gut microbiota, and then fermented to produce metabolites [3,4]. The variety and content of metabolites affects the variety and diversity of the gut microbiota, thus changing its structure. These metabolites and the gut microbiota bind to the corresponding receptors in intestinal epithelial tissue, thus affecting health.

*Glycyrrhiza uralensis* Fisch is a major medicinal material in China. Its roots and stems are commonly used as medicine and have the functions of nourishing the spleen and stomach and harmonizing hundreds of herbs. It was first recorded in “Shen Nong’s herbal classic” and “Compendium of Materia Medica” [5,6]. Polysaccharides, as one of the most abundant components in *Glycyrrhiza*, are characterized by antioxidation, antitumor, anticancer, antibacterial, anti-inflammatory, and antiviral properties and low cytotoxicity [7]. However, the current research has mainly focused on the extraction, purification, biological activities, and simple structural analysis of high-molecular-weight *Glycyrrhiza* polysaccharides [8,9]. In our previous studies, a low-molecular-weight (6.5 kDa) *Glycyrrhiza* polysaccharide (GP1, 200 mg/kg) was proven to improve the intestinal structure damage and immune disorder induced by cyclophosphamide and enhance immune regulation ability [10]. In addition, GP1 also promotes the proliferation of beneficial bacteria, such as *Lactobacillus*, *Muribaculum*, and *Lachnospiracear_NK4A136_group*, and the production of beneficial metabolites such as succinic acid, D-gluconic acid, D-galacturonic acid, and 4-pyridoxic acid. However, the toxicity of GP1 to immune cells and its immune enhancement effect on a healthy body remain unknown.

Herein, GP1’s effect on the proliferation of immune cells was analyzed via cell experiments to explore its immune enhancement effect on a healthy body, and the effect of the regulation of GP1 on the antioxidant capacity and immune response of mice was studied in vivo. The possible regulation mechanism of GP1 in enhancing the immune function of mice was analyzed using multi-omics.

## 2. Materials and Methods

### 2.1. Preparation and Structure of GP1

*Glycyrrhiza* polysaccharide (GP1) was extracted from *Glycyrrhiza uralensis* Fisch. (Emin, China) and purified using 2-diethylaminoethanol (DEAE) cellulose-52 (Solarbio, Beijing, China) and sephadex G-100 (Solarbio, China) columns. GP1’s molecular weight is 6.5 kDa, mainly composed of glucose, with the following backbone: →4)-α-D-Glc*p*-(1→, with branching at O-4 position of →4,6)-α-D-Glc*p*-(1→ mainly by α-D-Glc*p*-(1→ (Figures S1 and S2).

### 2.2. Antioxidant Capacity of GP1

#### 2.2.1. 1,1-Diphenyl-2-picrylhydrazyl (DPPH) Radical Scavenging

GP1 was prepared in different concentrations (1, 2, 3, 4, 5, 6 mg/mL) and vitamin C (Vc) was used as a positive control. According to the method in the literature [11,12], Vc and GP1 solution with different concentrations were added into DPPH ethanol solution, mixed evenly, stood for 30 min, and the absorbance was measured at 517 nm.

DPPH scavenging rate (%) = [1 − (A_sample_ − A_control_)/A_blank_] × 100

#### 2.2.2. 2,2′-Azino-bis3-ethylbenzothiazoline-6-sulfonic Acid) (ABTS) Radical Scavenging

Firstly, the ABTS (7 nM) and potassium persulfate (2.45 nM) solution were mixed, protected from light and left standing for 16 h. Subsequently, the ABTS solution was diluted with ultrapure water until its absorbance at 734 nm was 0.70 ± 0.02. Then, different concentrations of GP1 and Vc solution (1 mL) were mixed with ABTS solution (4 mL), left standing for 6 min, and the absorbance was measured at 734 nm [11,12].

ABTS scavenging rate (%) = (A_blank_ − A_sample_)/A_blank_] × 100

### 2.3. Cytotoxicity and Immunomodulatory Activity of GP1

#### 2.3.1. Cytotoxicity Analysis

The cells growing in logarithmic phase were removed, counted and inoculated into 96-well plates (5 × 10^4^ cells per well), and cultured in a refrigerator (5% CO_2_) at 37 °C for 24 h. According to the literature [13,14], lipopolysaccharide (LPS) was used as a positive control and phosphate buffer (PBS) was used as negative control, and then different concentrations of GP1 (5, 10, 25, 50, 100, 200, 500, 1000, 2000, and 5000 μg/mL) and FOS (1 μg/mL) solution were added and cultured for 24 h. The 96-well plate was washed twice with PBS, CCK-8 solution (10 μL) was added, and the absorbance at the absorbance of 450 nm was measured with microplate reader. The formula is as follows:

Cell proliferation rate (%) = (A_sample_ − A_blank_)/(A_control_ − A_blank_) × 100

#### 2.3.2. Phagocytic Activity

The experimental operation was as shown in Section 2.3.1, except that after cell culture, neutral red saline solution (Solarbio, China) (0.1%) was added, and after 1 h of being cultured, it was washed with PBS 3 times. After drying, glacial acetic acid and anhydrous ethanol (100 μL, 1:1, V:V) cell lysate were added to each well and left overnight at room temperature. The absorbance was measured at 570 nm by microplate reader.

#### 2.3.3. Determination of Nitric Oxide (NO) and Cytokines

The experimental operation method is as described in Section 2.3.1, except that after the intervention of GP1 and LPS, the supernatant was collected and processed according to the instructions of the NO, IL-1β, IL-6, and TNF-α ELISA kits (Elabscience, Wuhan, China) and the standard curve was drawn and the concentration was calculated.

### 2.4. Animal Experiment

#### 2.4.1. Animals and Experimental Process

All experimental procedures were performed per the Guide for the Care and Use of Laboratory Animals of the National Research Council. This study was approved by the Ethics Committee on Laboratory Animals of the First Affiliated Hospital of Shihezi University (A2023-098-01). Twenty-four male SPF mice, purchased from Henan Skobes Biotechnology Co., Ltd. (no. SCXK (Yu) 2020-0005, Anyang, China), were adapted for one week under specific conditions, and randomly divided into three groups: the control group (CON, normal saline: 0.1 mL/10 g), the low-dose GP1 group (GP1L: 200 mg/kg [10]), and the high-dose GP1 group (GP1H: 800 mg/kg, according to the results of cell experiments). The mice were given intragastric administration once a day for 8 weeks, and the changes in the basic physical characteristics of each group were recorded. After GP1 intervention for two hours on the last day, fresh feces were collected, frozen in liquid nitrogen, and stored in a refrigerator at −80 °C. After anesthesia, the neck was removed, and the organ tissues were collected, weighed, and stored in the refrigerator at −80 °C.

#### 2.4.2. Tissues’ Antioxidant Capacity

According to the kit instructions, fresh liver, spleen, thymus, kidney, heart, and lung tissues were mixed with a certain amount of extract, ground, and centrifuged, and the supernatants were collected to detect the contents of T-AOC, GSH-Px, SOD, and MDA in the tissues [10].

#### 2.4.3. Histopathological Analysis

The fresh intestine, spleen, and thymus tissues of mice were cleaned and placed in formalin solution, and immediately fixed in 10% formalin. Appropriate tissues were cut for paraffin embedding, sectionalization, dehydration, and staining with hematoxylin–eosin staining [10].

#### 2.4.4. Goblet Cell Number

Fresh colon tissue was fixed with 10% formalin solution, embedded in paraffin, sliced, and stained with Alcian Blue and Periodic Acid-Schiff (AB-PAS). The colon tissue structure and the number of goblet cells were observed under the microscope (Leica, Wetzlar, Germany), and the specific number of goblet cells in each area was calculated using Image J_v1.8.0 software (6 areas) [10].

#### 2.4.5. Immune Cell Number

The numbers of immune cells (T lymphocytes and macrophages) in the colon, spleen, and thymus tissues were measured using the immunofluorescence technique. After the tissue sections were dewaxed, the contents of T lymphocytes and macrophages were observed under an optical microscope using tissue sections incubated in primary antibodies (CD3 polyclonal antibody and F4/80 polyclonal antibody) and secondary antibodies (goat anti-rabbit-FITC) [10].

#### 2.4.6. Immune Factor Content

The collected blood samples were used to evaluate the effects of GP1 on cytokines. The contents of cytokines (IL-1β, IL-2, IL-4, IL-6, IL-10, IFN-γ, TGF-β3, and TNF-α), immunoglobulins (IgG and IgM), and chemokines (MIP-1α and MCP-1) in serum were detected according to the kit instructions [10].

#### 2.4.7. Gut Microbiota Structure Analysis

Total DNA was extracted from defrosted feces, quantified and purified using agarose gel electrophoresis, an ultraviolet spectrophotometer, and AMPure XT beads, and sequenced using a NovaSeq 6000 sequencing machine with 2 × 250 bp. The biodiversity, species composition and linear discriminant analysis effect size (LEfSe) were used to analyze the structure of the gut microbiota [10].

#### 2.4.8. Proteomic Analysis

After the fecal samples were thawed, urea and protease inhibitors were added to extract the protein. Then, the extracted protein was reduced, digested with trypsin, and desalted using a C18 column. LC-MS/MS analysis of tryptic peptides was conducted on a quadrupole Orbitrap mass spectrometer (Q Exactive HF-X, Thermo Fisher Scientific, Bremen, Germany) coupled to an EASY nLC 1200 ultra-high-pressure system (Thermo Fisher Scientific) via a nano-electrospray ion source [15]. The analysis conditions are shown in Table S1.

#### 2.4.9. Non-Targeted Metabonomics Analysis

After the fecal samples were thawed, the metabolites were extracted with a 50% methanol buffer. All sample analyses were performed using a Vanquish Flex UHPLC system (Thermo Fisher Scientific, Bremen, Germany) and an ACQUITY UPLC T3 column (100 mm × 2.1 mm, 1.8 µm, Waters, Milford, CT, USA). The mobile phase consisted of solvent A (water—0.1% formic acid) and solvent B (acetonitrile—0.1% formic acid) [15]. The analysis conditions are shown in Table S2.

### 2.5. Statistical Analysis

The experimental data were measured three times and are shown as means ± standard deviation (SD) unless otherwise noted. Different letters represent significant differences at *p* < 0.05. Multiple comparisons were performed using a one-way analysis of variance (ANOVA) followed by a least significant difference (LSD) post hoc test with the SPSS 26.0 software. * *p* < 0.05 and ** *p* < 0.01 were considered statistically significant in the diversity of gut microbiota.

## 3. Results

### 3.1. Antioxidant Activities of GP1

**Figure 1:**
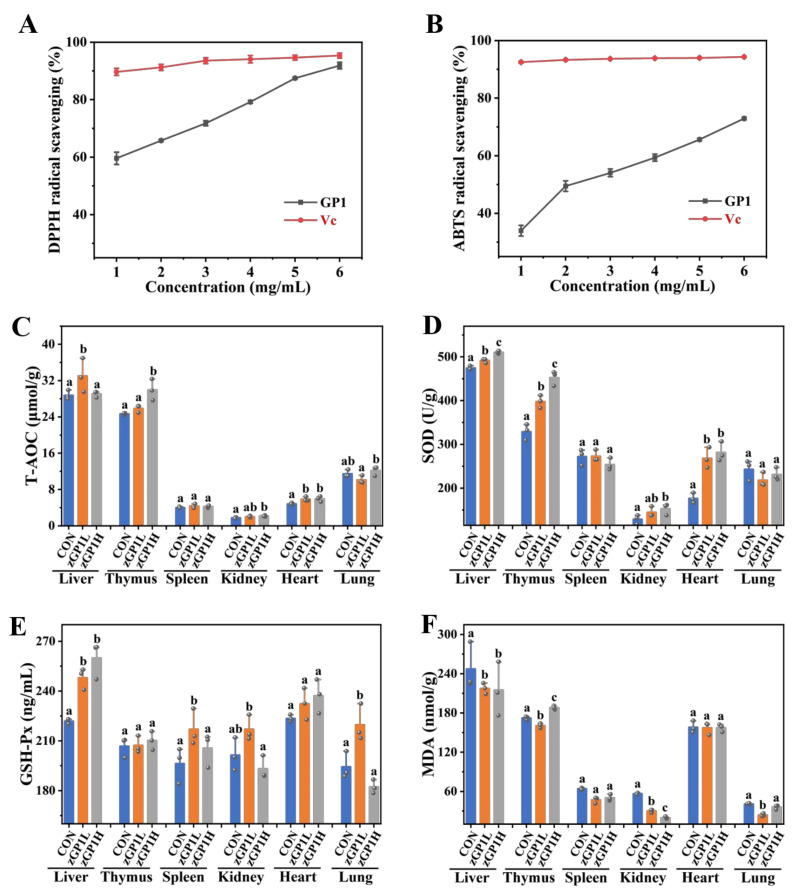
Antioxidant capacity of GP1. (**A**) DPPH radical scavenging. (**B**) ABTS radical scavenging. (**C**) The content of T-AOC. (**D**) The content of SOD. (**E**) The content of GSH-Px. (**F**) The content of MDA. The values are presented as mean ± SD, (*n* = 3). Different lowercase letters represent significant differences between the two groups (*p* < 0.05). Vc—vitamin C; CON—control group; GP1L—*Glycyrrhiza* polysaccharide low dose; GP1H—*Glycyrrhiza* polysaccharide high dose.

We studied GP1’s free radical-scavenging capacity to investigate whether it demonstrated antioxidant activity. DPPH and ABTS radicals are stable free radicals and are commonly used to determine antioxidant activities. The experimental results of DPPH and ABTS free radical scavenging by GP1 showed that GP1 displayed a remarkable effect on DPPH radical scavenging at an increased concentration (Figure 1A). At 6.0 mg/mL, GP1’s scavenging activity was 91.85 ± 1.14%, which was inferior to the antioxidant activity of L-ascorbic acid (95.33 ± 0.87%). Furthermore, GP1’s antioxidant activity was investigated via the ABTS radical-scavenging assay (Figure 1B). Similarly, the ABTS radical-scavenging activity of GP1 was enhanced at an increased concentration. GP1’s antioxidant capacity (72.92 ± 0.37%) increased outstandingly but was still weaker than that of Vc (94.32 ± 0.16%) (at 6.0 mg/mL). In vivo, GP1 intervention also significantly promoted the antioxidant capacity of mice organ tissues. Compared with the CON group, GP1L intervention in the GP1L group improved the contents of T-AOC, SOD, and GSH-Px in liver tissue by 12.96%, 3.46%, and 10.53% and reduced MDA by 12.06%; SOD increased by 17.34% and MDA decreased by 6.80% in thymus tissue, GSH-Px increased by 9.55% in spleen tissue, GSH-Px increased by 7.14% and MDA decreased by 46.44% in kidney tissue, SOD increased by 34.10% in heart tissue and GSH-Px increased by 11.52%, and MDA decreased by 41.74% in lung tissue (Figure 1C–F). Overall, the antioxidant capacity of GP1L was better than that of GP1H. These results show that GP1L had good antioxidant activity in vitro and in vivo, which may make it a potential therapeutic ingredient for excessive oxidation diseases.

### 3.2. Cytotoxicity and Immunomodulatory Activity of GP1 in RAW 264.7

**Figure 2:**
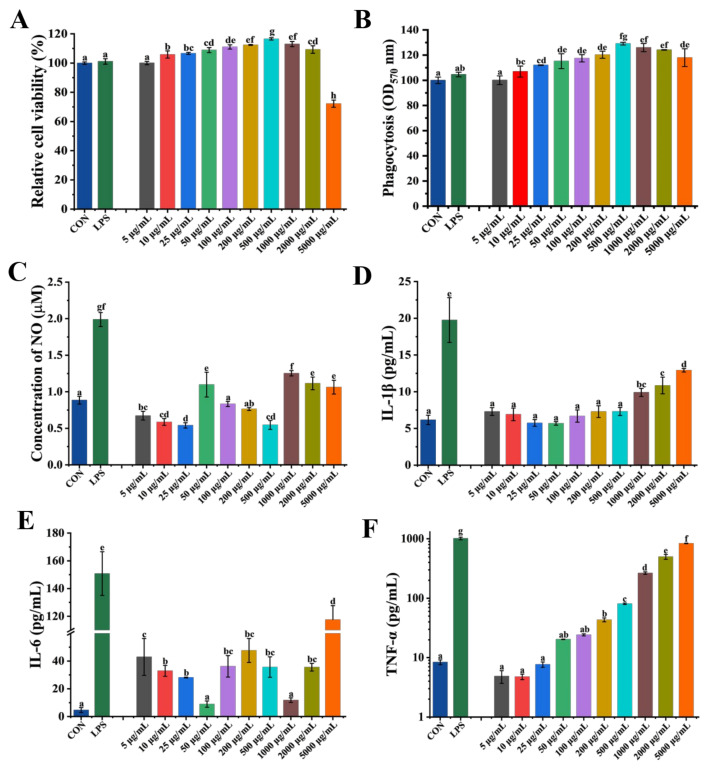
Effect of GP1 on macrophage RAW 264.7 activity. (**A**) Relative cell viability. (**B**) Phagocytosis activity. (**C**) The content of NO. (**D**) The content of IL-1β. (**E**) The content of IL-6. (**F**) The content of TNF-α. The values are presented as mean ± SD, (*n* = 3). Different lowercase letters represent significant differences between the two groups (*p* < 0.05). CON—control group; LPS—Lipopolysaccharide.

The CCK-8 and neutral red assays were used to evaluate GP1’s effect on RAW 264.7 macrophages to further explore its cytotoxicity and immunomodulatory activity. GP1 had no toxic effect even at a concentration of 2000 μg/mL and enhanced RAW 264.7 cells’ viability (Figure 2A). GP1 treatment enhanced the phagocytic ability of RAW 264.7 cells by 22.58% (500 μg/mL) compared with the CON group (Figure 2B).

The ELISA method was used to investigate the production of NO and cytokines in RAW264.7 cells after GP1 treatment. The production of NO by RAW264.7 cells increased by 19.37%, 29.36%, 20.62%, and 16.76% after treatment with 50, 1000, 2000, and 5000 μg/mL of GP1, while it decreased by 38.96% and 38.31% at 25 and 500 μg/mL of GP1, respectively (Figure 2C). Besides NO, activated RAW264.7 cells also promoted the secretion of some immune cytokines, including IL-1β, IL-6, and TNF-α. The levels of IL-1β increased in a concentration-dependent manner after treatment with GP1, increasing by 52.37% at 5000 μg/mL (Figure 2D). Moreover, IL-6 levels increased by 89.13%, 90.20%, and 96.03% at 5, 200, and 5000 μg/mL, respectively (Figure 2E). In addition, the level of TNF-α was similar to that in the LPS group treated with GP1 at 5000 μg/mL and increased in a concentration-dependent manner (Figure 2F). Thereby, the GP1 structure with →4)-D-Glc*p-*(1→ as the main residue had better immunomodulatory activity, making GP1 a potential ingredient in the treatment of diseases caused by immune disorders.

### 3.3. GP1 Enhances the Immunomodulatory Effect of Healthy Mice

**Figure 3:**
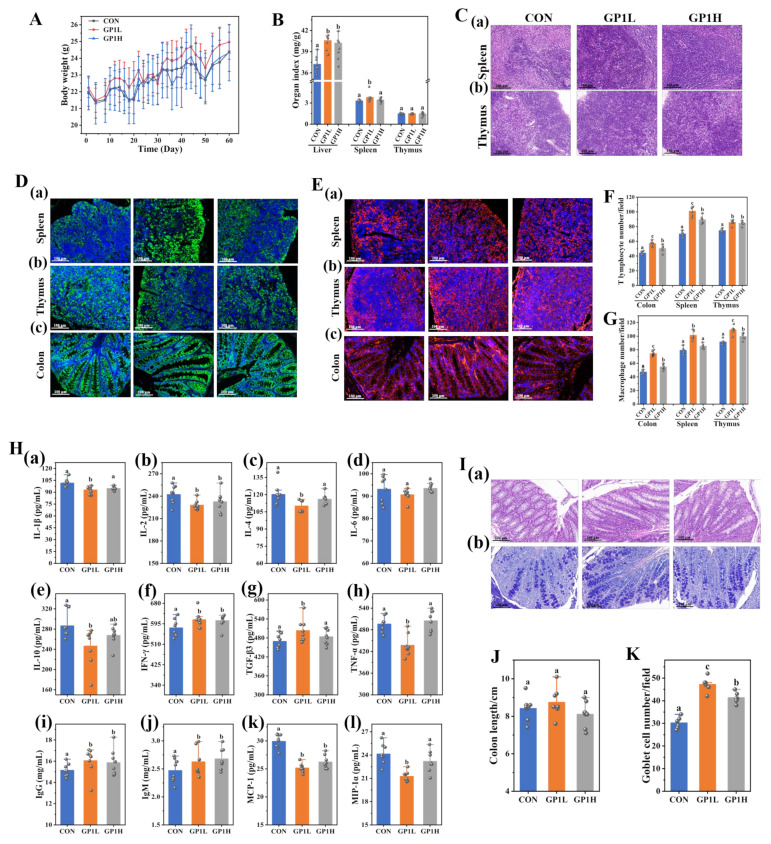
Immunoenhancement effect of GP1 on healthy mice. (**A**) Body weight of mice. (**B**) Immune organ indices. (**C**) Histopathological analysis of intestinal tissue: (**a**) structure of spleen; (**b**) structure of thymus. (**D**) Immunofluorescence representative images of T lymphocytes in tissues: (**a**) spleen tissue; (**b**) thymus tissue; (**c**) colon tissue. (**E**) Immunofluorescence representative images of macrophages in tissues: (**a**) spleen tissue; (**b**) thymus tissue; (**c**) colon tissue. (**F**) The number of T lymphocytes. (**G**) The number of macrophages. (**H**) The content of immune factors: (**a**) the content of IL-1β; (**b**) the content of IL-2; (**c**) the content of IL-4; (**d**) the content of IL-6; (**e**) the content of IL-10; (**f**) the content of IFN-γ; (**g**) the content of TGF-β3; (**h**) the content of TNF-α; (**i**) the content of IgG; (**j**) the content of IgM; (**k**) the content of MCP-1; (**l**) the content of MIP-1α. (**I**) Histopathological analysis of colon tissue: (**a**) H&E staining; (**b**) AB-PAS staining. (**J**) Colon length of mice. (**K**) Goblet cell number in intestinal tissue. The values are presented as mean ± SD, (*n* = 3). Different lowercase letters represent significant differences between the two groups (*p* < 0.05). CON—control group; GP1L—*Glycyrrhiza* polysaccharide low dose; GP1H—*Glycyrrhiza* polysaccharide high dose.

We analyzed the basic physical characteristics of mice, the structural changes in the intestinal tract and immune organs, immune cell number, and cytokines, to study the immune enhancement effect of GP1 on healthy mice. GP1 intervention improved the body weight and immune organ index and increased the index of the liver and spleen by 8.27% and 13.00%, respectively (Figure 3A,B). The spleen is the largest lymphatic organ in the human body; it provides defense for the body and enhances immunity. The thymus is the main place where immune cells mature and develop. GP1 improved the structure of the spleen and thymus, increased the number of immune cells, and enhanced the body’s immunity (Figure 3C). Immune cells are the main cells of the immune organs to exert immune regulation. GP1 significantly promoted the number of T lymphocytes (30.58% and 12.87%, respectively) and macrophages (21.75% and 16.18%, respectively) in the spleen and thymus (Figure 3D(a,b),E(a,b),F,G). Cytokines are a kind of small molecular protein with a wide range of biological activities that are synthesized and secreted by immune cells (macrophages and T lymphocytes) and some non-immune cells (endothelial cells) [12]. GP1 intervention promoted the secretion of IFN-γ, TGF-β3, IgG, and IgM by 5.55%, 6.76%, 5.65%, and 5.33%, respectively, and decreased the contents of cytokines such as IL-1β, IL-2, IL-4, IL-10, and TNF-α by 8.57%, 5.91%, 8.41%, 13.96%, and 11.87%, respectively, and chemokines such as MCP-1 and MIP-1 by 15.86% and 11.92%, respectively. The content of IL-6 did not significantly change (Figure 3H).

The intestine is the largest immune organ in the human body [13]. GP1 increased the villi length and density and improved the intestinal structure (Figure 3I(a),J). Meanwhile, GP1 significantly increased the number of goblet cells by 36.04% and increased the secretion of mucin and intestinal barrier function (Figure 3I(b),K). The immunofluorescence results show that GP1 increased the number of T lymphocytes and macrophages in colon tissue by 22.58% and 36.55%, respectively (Figure 3D(c),E(c),F,G). These results show that GP1 improved the tissue structure, increased the number of immune cells and goblet cells, and promoted the secretion of cytokines, immunoglobulins, and mucins, thus enhancing the body’s intestinal barrier and immune response function. In addition, the immunomodulatory effect of 200 mg/kg of GP1 on mice was better than 800 mg/kg, which indicates that the daily intake of GP1 should not be too high.

### 3.4. GP1 Optimizes the Composition Structure of Gut Microbiota

**Figure 4:**
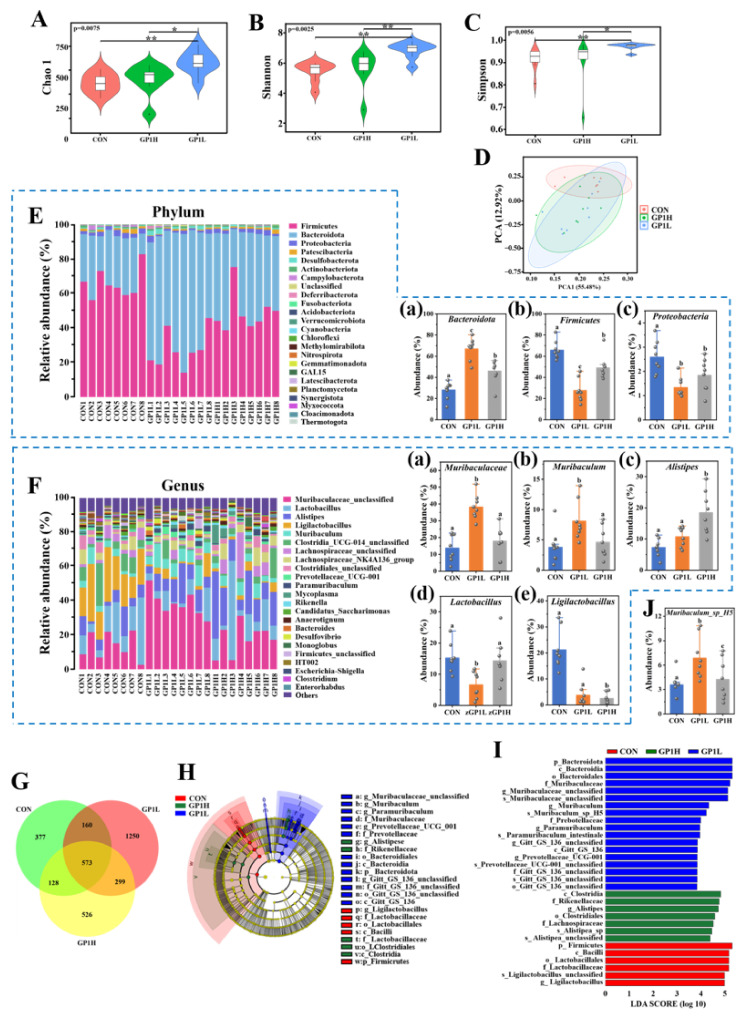
Effect of GP1 on gut microbiota structure of healthy mice. (**A**) Diversity analysis of gut microbiota: Chao 1 (*p* = 0.0075). (**B**) Diversity analysis of gut microbiota: Shannon (*p* = 0.0025). (**C**) Diversity analysis of gut microbiota: Simpson (*p* = 0.0056). (**D**) β-diversity. (**E**) Composition of gut microbiota (phylum): (**a**) abundance of Bacteroidota; (**b**) abundance of Firmicutes; (**c**) abundance of Proteobacteria. (**F**) The composition of gut microbiota (genus): (**a**) abundance of *Muribaculaceae*; (**b**) abundance of *Muribaculum*; (**c**) abundance of *Alistipes*; (**d**) abundance of *Lactobacillus*; (**e**) abundance of *Ligilactobacillus*. (**G**) Venn diagram. (**H**,**I**) LEfSe analysis. (**J**) Abundance of *Muribaculum_sp_H5*. The values are presented as means ± SD (*n* = 3). Different lowercase letters represent significant differences between the two groups (*p* < 0.05); * *p* < 0.05 and ** *p* < 0.01. CON—control group; GP1L—*Glycyrrhiza* polysaccharide low dose; GP1H—*Glycyrrhiza* polysaccharide high dose.

The complex and diverse gut microbiota affects the physiological functions of a human host, such as digestion and absorption, energy metabolism, and immune defense, by regulating nerves, immunity, and the endocrine system. Therefore, genome analyses were used to analyze the composition of intestinal microorganisms in the feces to study the change in the gut microbiota structure and its influence on the body after GP1 intervention. These sequencing results show that the Chao 1, Shannon, and Simpson indexes of the gut microbiota increased by 37.66%, 20.70%, and 6.14% (GP1L; *p* < 0.05) and 9.73%, 3.66%, and 0.1% (GP1H; *p* > 0.05), respectively, after 8 weeks of intervention with GP1 (Figure 4A–C). Compared with the CON group, the community structure of the GP1 group was significantly separated, forming a new flora structure (Figure 4D). At the phylum level, oral GP1L and GP1H levels increased the abundance of Bacteroides by 58.18% and 39.00%, respectively, and decreased Firmicutes by 58.15% and 25.50% and Proteobacteria by 48.54% and 28.98%, respectively (Figure 4E). At the genus level, the abundances of beneficial bacteria significantly increased after intervention with GP1, including *Muribaculaceae_unclassified*, *Lactobacillus*, *Alistipes*, *Ligilactobacillus*, and *Muribaculum* (Figure 4F). Compared with the CON group, the abundances of *Muribaculaceae_unclassified* and *Muribaculum* in the GP1L group increased by 64.14% and 53.45%, respectively, that of *Alistipes* in the GP1H group increased by 60.64%, and that of *Ligilactobacillus* in the GP1L and GP1H groups all decreased by 82.38% and 88.43%, respectively. At the species level, the abundance of *Muribaculum_sp_H5* increased by 47.77% (Figure 4F). These results show that the abundance of obligate anaerobic bacteria increased and that of facultative anaerobic bacteria decreased after intervention with GP1, which should be related to GP1’s enhancement of antioxidant capacity.

A Venn diagram was used to analyze the unique and common gut microbiota in the feces of the mice in each group. The results show that there were 377 unique bacteria in the CON group, 1250 in the GP1L group, and 526 in the GP1H group (Figure 4G). Subsequently, the differential bacteria of the gut microbiota among the three groups were compared via LEfSe analysis (Figure 4H,I). The results show that there were 5 species of differential bacteria with LDA > 4 in the CON group, including Firmicutes and *Bacilli*; 12 species in the GP1L group, including *Muribaculaceae_unclassified*, *Muribaculum*, *Prebotellaceae_UCG_001*, and *Paramuribaculum*; and 6 species in the GP1H group, including *Clostridia*, *Rikenellaceae*, and *Aalistipses*. The results show that the diversity, abundance, and species composition of the gut microbiota in the GP1L and GP1H groups were quite different. Moreover, the proliferation effect of prebiotics in the GP1L group was significantly better than that in the GP1H group, thereby optimizing the structure of the gut microbiota.

### 3.5. GP1 Changes Expression of Functional Proteins in Gut Microbiota

**Figure 5:**
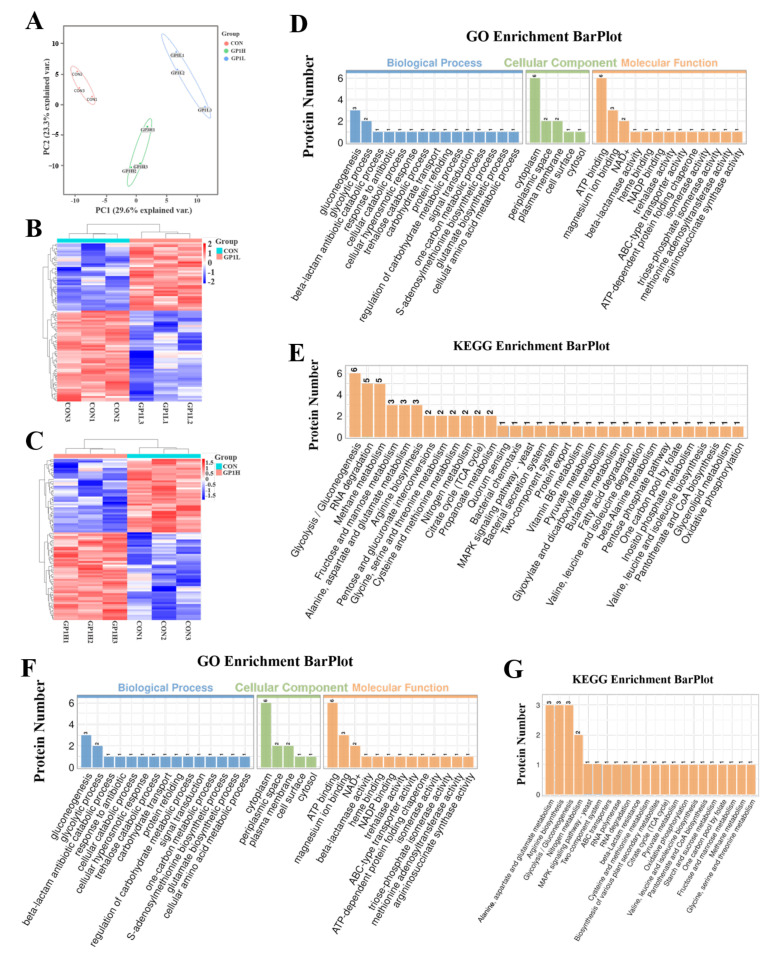
Functional prediction and pathway enrichment of differential proteins in the gut microbiota treated with GP1 and saline. (**A**) PCA analysis of proteins. (**B**) Differential protein—GP1L group. (**C**) Differential protein—GP1H group. (**D**) Function prediction of differential protein by GO–GP1L. (**E**) Pathway enrichment of differential protein by KEGG–GP1L. (**F**) Function prediction of differential protein by GO–GP1H. (**G**) Pathway enrichment of differential protein by KEGG–GP1H. CON—control group; GP1L—*Glycyrrhiza* polysaccharide low dose; GP1H—*Glycyrrhiza* polysaccharide high dose.

The metaproteomics results show that there were 87 functional proteins with significant differences in the GP1L group and 90 in the GP1H group compared with the CON group (Figure 5A–C and Figure S3A–F, and Tables S3 and S4), as well as the main biological processes, including glycolytic process and gluconeogenesis, and metabolic pathways, including glycolysis/gluconeogenesis, RNA degradation, methane metabolism, fructose and mannose metabolism, starch and sucrose metabolism, the MAPK signaling pathway, vitamin B6 metabolism, alanine, aspartate and glutamate metabolism, arginine biosynthesis, pentose and glucuronate interconversions, glycine, serine and threonine metabolism, cysteine and methionine metabolism, nitrogen metabolism, the TCA cycle, and propanoate metabolism (Figure 5D–G). These results show that these differential functional proteins in the gut microbiota were mainly used to regulate and activate the intracellular glucose metabolism pathway and immunomodulatory pathway, similar to the previous experimental results in the immunosuppression model.

### 3.6. GP1 Promotes Health by Producing Beneficial Metabolites

**Figure 6:**
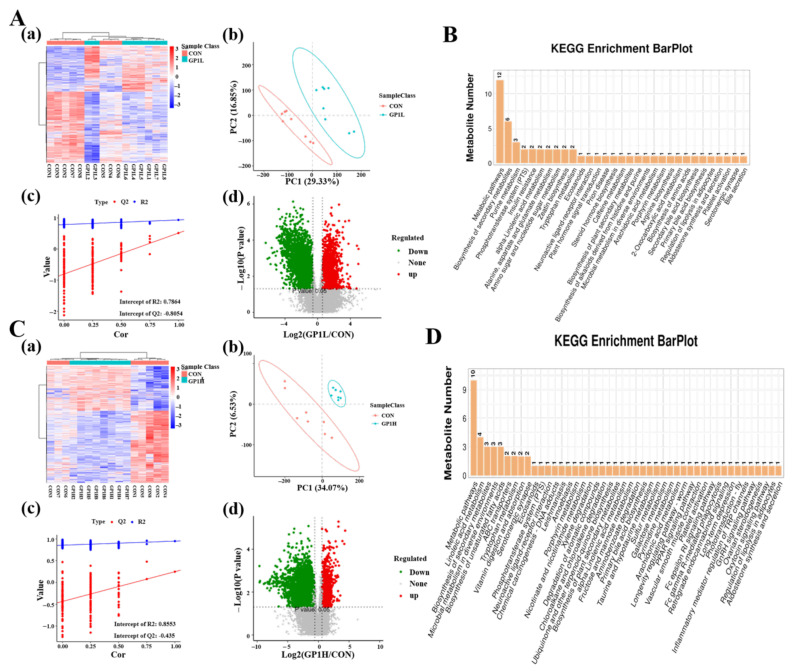
Differential metabolites in feces and their pathway enrichment. (**A**) Differential metabolites—GP1L group: (**a**) Heatmap; (**b**) PCA; (**c**) PLS-DA; (**d**) Volcano map. (**B**) Pathway enrichment of differential metabolites—GP1L group. (**C**) Differential metabolites—GP1H group: (**a**) Heatmap; (**b**) PCA; (**c**) PLS-DA; (**d**) Volcano map. (**D**) Pathway enrichment of differential metabolites—GP1H group. CON—control group; GP1L—*Glycyrrhiza* polysaccharide low dose; GP1H—*Glycyrrhiza* polysaccharide high dose.

We detected the metabolites in mice feces via non-targeted metabonomics to find the metabolites produced via these metabolic pathways regulated by different functional proteins of the gut microbiota. The results show that 38,420 and 36,097 samples were detected in the GP1L and GP1H groups, respectively (Figure S4A–F). Conditional screening (ratio ≥ 2 or ratio ≤ 1/2; *p* < 0.05; and VIP ≥ 1) revealed that there were 19 significantly different metabolites in the GP1L group, including niacin, α-tocopherol, stearidonic acid, D-sorbitol 6-phosphate, 5-hydroxyindole-3-acetic acid, taurine, melibiose, and arachidonic acid (Figure 6A), and 16 in the GP1H group, including colnelenic acid, corticosterone, glucosamine 6-phosphate, traumatic acid, and D-glucosamine-6-phosphate (Figure 6C). By comparison, the metabolites of different doses of GP1 were completely different after being degraded by the gut microbiota, and the beneficial effects of GP1L were better than those in the GP1H group. These results show that GP1 has a beneficial effect on the body in a certain concentration range, which was consistent with GP1’s immunomodulatory activity.

The results of KEGG pathway enrichment show that the metabolic pathways involved in the differential metabolites in the GP1H group mainly included metabolic pathways, secondary metabolites metabolism, α-linolenic acid metabolism, amino sugar and nucleotide sugar metabolism, zeatin biosynthesis, tryptophan metabolism, primary bile acid synthesis, and bile secretion (Figure 4B). Besides these pathways, in the GP1L group, the metabolic pathways included ABC transporter, vitamin digestion and absorption, neuroactive ligand–receptor interaction, hydrochloride and nicotinamide metabolism, taurine and hypotaurine metabolism, galactose metabolism, the PPAR signaling pathway, arachidonic acid metabolism, the FcεRI signaling pathway, FcγR-mediated phagocytosis, inflammatory mediator regulation of the TRP channel, and the GnRH signaling pathway (Figure 4D).

### 3.7. Prebiotic Effect of GP1L

**Figure 7:**
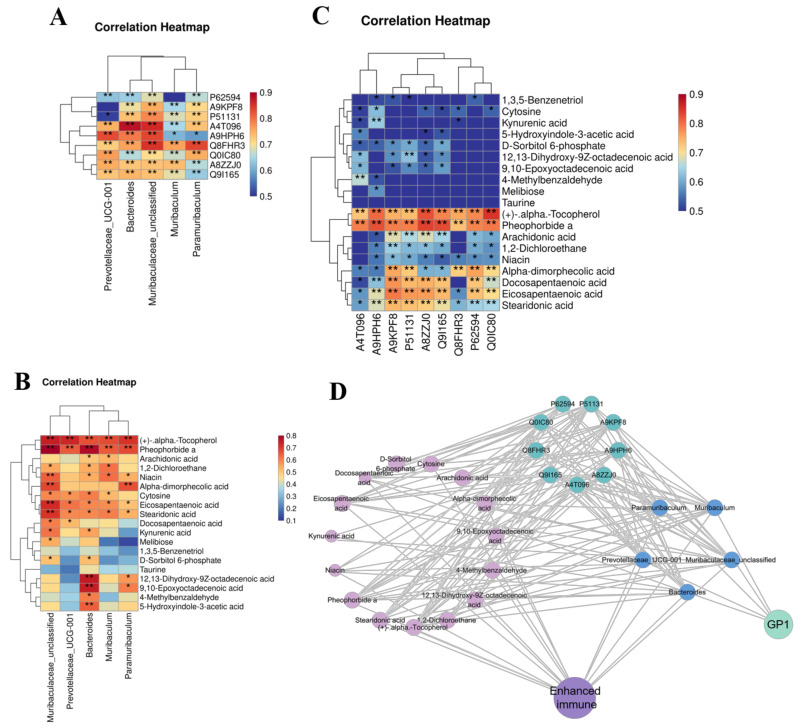
Relationship diagram of beneficial effects of GP1. (**A**) The correlation between dominant flora and differential proteins. (**B**) The correlation between dominant flora and differential metabolites. (**C**) The correlation between differential metabolites and differential proteins. (**D**) Probiotic pathway of GP1 to healthy. The light green circle represents GP1, the blue circle represents GP1L-dominant flora, the green circle represents differential protein, the light purple represents characteristic metabolites, and purple represents immune system. * *p* < 0.05 and ** *p* < 0.01. GP1—*Glycyrrhiza* polysaccharide 1.

Spearman correlation analysis was carried out on the differential bacteria, functional proteins, and metabolites obtained in the previous screening to explore the possible mechanism of probiotics of GP1L. GP1L-dominant bacteria, such as *Prevotellaceae_UCG-001*, *Bacteroides*, *Muribacelaceae_unclassified*, *Muribaculum*, and *Paramuribaculum*, were positively correlated with differential functional proteins, including β-lactamase TEM, ketoacid reductase isomerase (NADP), ATP-binding protein YcjV, chaperone GroEL 2, 2,3-phosphoglycerate-dependent phosphoglycerate mutase, formate–tetrahydrofolate ligase, periplasmic trehalase, triose-phosphate isomerase, and cytochrome b/c1 (Figure 7A). They were also positively correlated with differential metabolites including α-tocopherol, pheophorbide A, arachidonic acid, nicotinic acid, α-diphosphate, cytosine, eicosapentaenoic acid, stearenoic acid, 12,13-dihydroxy-9Z-octadecenoic acid, 9,10-epoxyectadecenoic acid, 5-hydroxyindole-3-acetic acid, and docosapentaenoic acid (Figure 7B). Differential functional proteins, including β-lactamase TEM, ketoacid reductase isomerase (NADP), chaperone GroEL 2, phosphoglycerate mutase, formate–tetrahydrofolate ligase, periplasmic trehalase, triose-phosphate isomerase, and cytochrome b/c1 were positively correlated with differential metabolites including α-tocopherol, pheophorbide A, arachidonic acid, nicotinic acid, α-diphosphate, eicosapentaenoic acid, and docosapentaenoic acid (collectively referred to as postbiotics) (Figure 7C).

Spearman correlation was used to analyze the beneficial effects of dominant bacteria and postbiotics induced by GP1L on the body. Figure 7D shows that GP1L induced the proliferation of probiotics including *Prevotellaceae_UCG-001*, *Bacteroides*, *Muribacelaceae_unclassified*, *Muribaculum*, and *Paramuribaculum* and upregulated the expression of functional proteins related to sugar metabolism, thereby increasing the production of metabolites such as α-tocopherol, pheophorbide A, arachidonic acid, nicotinic acid, α-diphosphate, eicosapentaenoic acid, and docosapentaenoic acid. These probiotics and postbiotics cooperate to improve the body’s defense, thus achieving a beneficial effect. The prebiotic effect of 200 mg/kg of GP1 was better than that of 800 mg/kg.

## 4. Discussion

Some bad habits, including long-term irregular life patterns (such as staying up late), chemotherapy drugs (such as cyclophosphamide, etc.), abuse of antibiotics, depression or nervousness, lack of exercise, and heavy drinking or smoking will destroy the gut microbiota, which leads to immune disorders and various diseases, such as obesity, diabetes, inflammatory bowel disease, etc. [14,16]. The low-molecular-weight GP1 isolated in our laboratory had the functions of improving the antioxidant capacity of tissues, enhancing the body’s immune defense capacity, improving the structure of the gut microbiota, promoting the proliferation of probiotics, and increasing the production of beneficial metabolites, and was, thus, expected to become a prebiotic with extensive activities.

Previous studies have shown that GP1 improved intestinal health, maintained the homeostasis of the gut microbiota, and enhanced immune regulation ability by increasing the proliferation of beneficial bacteria and the production of postbiotics. However, the beneficial effect of GP1 on a normal body has not been explored. In this study, the antioxidant capacity, immunomodulation capacity, gut microbiota composition, and beneficial mechanism of healthy mice were detected after intervention with GP1 for a period of time. The intervention results show that GP1L increased the liver index, spleen index, and colon length of healthy mice, reduced the secretion of pro-inflammatory cytokines, including IL-1β, IL-2, IL-4, IL-10, and TNF-α, and chemokines, including MIP-1α and MCP-1, and promoted the production of IFN-γ, TGF-β3, IgG, and IgM, thus enhancing the body’s immune regulation ability. Wang et al. found that polysaccharides extracted from *Hericium erinaceus* improved the intestinal health of healthy mice [17]. Huang et al. also observed that those active polysaccharides enhanced the antioxidant capacity, immunoregulatory activity, and antitumor effects, reduced inflammation, and regulated the blood sugar and blood lipid levels of healthy bodies [18]. However, the prebiotic effect of GP1H (800 mg/kg) was significantly reduced compared with GP1L (200 mg/kg), consistent with the results of cell experiments. One of GP1L’s beneficial mechanisms is to enhance the body’s immune regulation ability.

Intervention with GP1L can also affect the secretion of antioxidant factors (e.g., T-AOC, SOD, and GSH-Px) in the tissues of healthy mice and regulate the body’s antioxidant capacity. T-AOC is the total antioxidant level composed of various antioxidant substances and antioxidant enzymes and can be used to protect cells and the body from oxidative stress damage caused by reactive oxygen free radicals [19]. GP1L significantly increased the concentration of T-AOC in various organ tissues and enhanced tissues’ antioxidant capacity. In addition, GP1L increased the contents of SOD and GSH-Px in tissues, which could transform reactive oxygen species into harmless substances, thus protecting cells from oxidative damage [20]. Chen et al. found that *cushaw* polysaccharide containing α-glycosidic bonds demonstrates good free radical scavenging activity [21]. Polysaccharides extracted from the root of *Chuanminshen violaceum* (CVPS) can scavenge DPPH, hydroxyl, and superoxide anion radicals, increase SOD and CAT levels, and decrease MDA content [22]. Moreover, the antioxidant activity of GP1L is better than that of CVPS. MDA is a product of lipid peroxidation that can cause the cross-linking polymerization of macromolecules such as proteins and nucleic acids and displays cytotoxicity [23]. GP1L significantly reduced MDA levels in various organ tissues. The antioxidant capacity in the GP1L group was better than that in the GP1H group. These results show that GP1L can play a beneficial role by increasing the antioxidant capacity of organs in the body, promoting cell regeneration, and then improving health.

When GP1 was transported to the intestine, it promoted the proliferation of beneficial bacteria, such as *Muribaculaceae_unclassified*, *Muribaculum*, *Prevotellaceae_UCG-001*, *Paramuribaculum*, and *Bacteroides*, all belonging to Bacteroides (GP1L). *Muribaculaceae_unclassified* is an anaerobic intestinal symbiotic bacterium, which has many functions, including inhibiting the proliferation of pathogenic bacteria, reducing the intestinal inflammation and tissue damage of the host, decomposing complex polysaccharides in food, producing beneficial metabolites, promoting intestinal mucosal health, and increasing intestinal peristalsis and absorption. In addition, it can prevent obesity and metabolic diseases by regulating host energy metabolism, which is crucial for intestinal homeostasis and host health [24]. *Muribaculum* is a characteristic degradation bacterium of GP1, which has the functions of producing beneficial metabolites, promoting intestinal health, and enhancing host immune regulation [25]. *Prevotellaceae_UCG-001* maintains intestinal homeostasis and enhances intestinal barrier function and the host immune defense ability by producing antibiotics, inhibiting the proliferation of harmful bacteria, metabolizing carbohydrates, regulating intestinal nutritional balance, secreting signal molecules, and promoting the growth and activation of immune cells [26]. *Bacteroides* is a kind of obligate anaerobic bacteria that contains various degrading enzymes. It is used to metabolize polysaccharides and cellulose, to produce beneficial metabolites, such as SCFAs, to induce intestinal epithelial cells to secrete IgA, and to cooperate with other bacteria to maintain intestinal homeostasis and intestinal inflammation and inhibit the colonization of harmful bacteria [27]. Zhu et al. found that *turmeric* polysaccharides promoted the proliferation of *Lactobacillus* and *Bacteroides* in the intestine of mice [28]; *algal* polysaccharides increased the abundance of *Bacteroidia*, *Bacilli*, *Clostridia*, and *Verrucomicrobia* [29] and *Nigella sativa* seed polysaccharides mainly promoted the proliferation of *Muribaculaceae_Unclassified* and *Bacteroides* [30], indicating that different kinds of plant polysaccharides induce different kinds of intestinal characteristic bacteria. However, in the GP1H group, the abundance of *Alistipes* and *Rikenella* (both belonging to *Rikenellaceae*) was mainly increased, which could regulate the balance of the gut microbiota, increase intestinal peristalsis, and promote the absorption of nutrients [31]. Combined with the above results, it was found that GP1L regulated the intestinal health of the host and enhanced its immune regulation function by increasing the proliferation of intestinal probiotics and promoting the production of beneficial metabolites and signal molecules. Hence, these bacteria derived from GP1L intervention have beneficial effects on intestinal health and the immune system. However, the regulatory effect of GP1L on the gut microbiota structure was significantly better than that of GP1H, indicating that GP1 had a more significant beneficial effect on the body in a certain concentration range. *Longan pulp* polysaccharide (LP) regulated the abundance of *Lactobacillus*, *Pediococcus*, and *Bifidobacterium* [32]. *Flammulina velutipes* polysaccharide (FVP) increased the richness of *Bacteroides* and inhibited the proliferation of *Desulfovibrionales* and *Clostridium* [33]. Yang et al. found that *Hericium erinaceus* polysaccharides (HEPs) influenced the abundance of *Lachnospiraceae*, *Akkermansiaceae*, *Rikenellaceae*, and *Bacteroidaceae* [34]. Additionally, *pumpkin* polysaccharide (PP) increased the abundance of *Bacteroidetes*, *Prevotella*, *Deltaproteobacteria*, *Oscillospira*, *Veillonellaceae*, *Phascolarctobacterium*, *Sutterella*, and *Bilophila* [35]. The types of gut microbiota induced by GP1L have better beneficial effects on the body compared with LP, FVP, HEPs, and PP.

After ingesting GP1L, the structure of the gut microbiota changed, and then the types of postbiotics were changed. The different functional proteins and postbiotics in the gut microbiota were analyzed, revealing that the contents of niacin, α-tocopherol, stearidonic acid, 9,10-epoxyoctadecenoic acid, D-sorbitol 6-phosphate, 5-hydroxyindole-3-acetic acid, taurine, docosapentaenoic acid, melibiose, arachidonic acid, and eicosapentaenoic acid in the GP1L group significantly increased. D-sorbitol 6-phosphate is a naturally occurring polyol with the functions of antioxidation, anti-inflammation, heart protection, and immune system regulation. Additionally, it increases the amount of intestinal fluid and intestinal peristalsis, improves intestinal function, and relieves constipation [36]. Stearidonic acid, arachidonic acid, eicosapentaenoic acid, and docosapentaenoic acid are all fatty acids with the functions of regulating metabolism, improving immune response, promoting intestinal peristalsis, anti-inflammatory, antioxidant, and antibacterial effects, and lowering blood pressure [37,38,39,40]. Melibiose is a kind of sugar dimer exhibiting the functions of regulating macrophage phagocytosis, leukocyte activity, and sterilization [41]. Taurine is a sulfur-containing non-protein amino acid that can promote infant brain tissue development, protect vision, reduce the occurrence of cardiovascular diseases, enhance the body’s immune ability, and relieve fatigue [42]. Niacin and α-tocopherol are vitamins with the functions of scavenging free radicals in vivo, improving skin health, promoting the proliferation and differentiation of immune cells, enhancing immunity, reducing blood lipid levels, preventing atherosclerosis and thrombosis, and reducing the occurrence of myocardial infarction and cerebral infarction [43,44]. However, the metabolites in the GP1H group significantly increased, mainly glucosamine 6-phosphate and *N*-acetyl-L-glutamic acid. Glucosamine 6-phosphate can inhibit tumor factors and autoimmune antigens and has a good anti-inflammatory effect [45]. *N*-acetyl-L-glutamic acid is an important amino acid with the functions of protecting liver health, reducing fatigue, promoting intestinal mucosal repair, and maintaining intestinal health [46]. *Ascophyllum nodosum* polysaccharide produced characteristic metabolites such as betaine, L-carnitine, and aminoimidazole carboxamide ribonucleotide by regulating microorganisms [47]. Polysaccharides from *Cordyceps militaris* increased the contents of brassicasterol and 4′-O-methylkanzonol W [48], which indicated that the types of intestinal characteristic metabolites were related to the plant polysaccharides and intestinal characteristic flora. The GP1L group had more kinds of postbiotics than the GP1H group, promoting health and immunity. This indicates that GP1L also produced significant beneficial characteristic metabolism in a normal body, thus improving the body’s intestinal environment, metabolic function, and antioxidant and immune ability.

## 5. Conclusions

In conclusion, GP1 enhanced the immune function of mice by increasing the index of immune organs and the contents of immune cytokines and immunoglobulins. It also promoted the secretion of T-AOC, GSH-Px, and SOD in various organs and tissues, reduced the MDA content, enhanced the body’s antioxidant capacity, promoted cell proliferation, and prolonged life. In addition, it promoted the proliferation of beneficial bacteria, including *Muribaculaceae_unclassified*, *Muribaculum*, and *Prevotellaceae_UCG-001*, and the production of postbiotics, including α-tocopherol, arachidonic acid, melibiose, taurine, and nicotinic acid, thus enhancing the immune response ability of healthy mice and achieving prebiotic effects. It is worth noting that the concentration of GP1 should not be too high, otherwise it will be counterproductive. However, the specific mechanism of action and the effector cells and receptors in the intestine remain unexplored. In addition, functional products with low-molecular-weight GP1 as the main component need further research and development.

## Data Availability

Beneficial effects of low molecular weight GP1 on healthy data were deposited at Mendeley Data (https://data.mendeley.com/, accessed on 20 September 2024) with Reserved DOI: 10.17632/mshv5vp83c.1. Source data are provided with this paper.

## Supplementary Materials

The following supporting information can be downloaded at: https://www.mdpi.com/article/10.3390/nu17020225/s1, Figure S1 Purification, basic properties and glycoside bond analysis of GP1. (The article on structural analysis of GP1 is being published.). Figure S2 Structural analysis of GP1. (The article on structural analysis of GP1 is being published.); Figure S3 Detection and analysis of differential proteins in gut microbiota. (A) Overall deviation statistical chart. (B) Statistical diagram of peptide length. (C) Statistical chart of the number of peptide segments matched by protein. (D) Identification of isoelectric point and molecular weight scatter plot of protein. (E) Global analysis clustering heat map. (F) Sample correlation matrix diagram; Figure S4 Detection and identification of metabolites in the fecal treated with GP1 and saline. (A) Total Ion Chromatogram – GP1L; (a) pos; (b) neg. (B) Metabolites m/z-rt distribution – GP1L: (a) pos; (b) neg. (C) Classification of metabolites – GP1L. (D) Total Ion Chromatogram – GP1L; (a) pos; (b) neg. (E) Metabolites m/z-rt distribution – GP1L: (a) pos; (b) neg. (F) Classification of metabolites – GP1L. Table S1 Liquid chromatographic gradient; Table S2 Gradient elution condition; Table S3 Differential protein in GP1L group; Table S4 Differential protein in GP1H group.

## Abbreviations

Abbreviations	Full Name
GP	*Glycyrrhiza* polysaccharide
T-AOC	Total antioxidant capacity
GSH-Px	Glutathione peroxidase
SOD	Superoxide dismutase
MDA	Malondialdehyde
DPPH	2,2-Diphenyl-1-picrylhydrazyl
ABTS	2,2′-azino-bis(3-ethylbenzothiazoline-6-sulfonic acid)
LPS	Lipopolysaccharide
PBS	Phosphate-Buffered Saline
CCK-8	Cell Counting Kit-8
NO	Nitric Oxide
IL-1β	Interleukin 1β
IL-2	Interleukin 2
IL-4	Interleukin 4
IL-6	Interleukin 6
IL-10	Interleukin 10
IFN-γ	Interferon-gamma
TGF-β3	Transforming growth factor-β3
TNF-α	Tumor necrosis factor α
IgG	immunoglobulin G
IgM	immunoglobulin M
MIP-1α	Macrophage Inflammatory Protein-1 alpha
MCP-1	Monocyte chemoattractant protein-1
KEGG	Kyoto encyclopedia of genes and genomes
GO	Gene ontology
PLS-DA	Partial Least Squares Discriminant Analysis
