# Supplementary material for: Glycyrrhiza uralensis Polysaccharide Modulates Characteristic Bacteria and Metabolites, Improving the Immune Function of Healthy Mice
Source: Nutrients. 2025 Jan 9;17(2):225. doi: 10.3390/nu17020225 (PMC11767424; doi:10.3390/nu17020225)
Supplement: Supplementary file 1 [file nutrients-17-00225-s001.zip › nutrients-3376523-supplementary.pdf]

Glycyrrhiza uralensis Polysaccharide Modulates Characteristic Bacteria and Metabolites, Improving the Immune Function of Healthy Mice

Wangdi Song [1,2], Taifeng Zhang [3], Yunyun Wang [1], Shengnan Xue [1], Yan Zhang [1,*] and Genlin Zhang [1,*]

[1] State Key Laboratory Incubation Base for Green Processing of Chemical Engineering, School of Chemistry and Chemical Engineering, Shihezi University, Shihezi 832003, China; swd_pharmacy@163.com (W.S.); wyy2021090130@163.com (Y.W.); xsn99805@163.com (S.X.)
[2] Key Laboratory of Xinjiang Endemic Phytomedicine Resources Ministry of Education, College of Pharmacy, Shihezi University, Shihezi 832003, China
[3] Testing Center of Xinjiang Tianye Co., Ltd., Shihezi 832099, China; swdztf228519@163.com
* Correspondence: zyly605@163.com (Y.Z.); zhanggl_bce@shzu.edu.cn (G.Z.)

Supplementary data:

Table S1. Liquid chromatographic gradient.

Time（min）	Mobile phase B ratio（%）
00	8
05	12
38	30
45	40
60	95

Table S2. Gradient elution condition

Time（min）	Mobile phase B ratio（%）
00	5
0.5	5
7	100
8	100
8.1	100
10	5

Table S3. Differential protein in GP1L group.

ID	Accession	Gene Name	Description	FC	pValue	GO	KEGG
BLAT_SALTI	P62594	bla	Beta-lactamase TEM	25.83967	0.000339	GO:0008800;GO:0030655;GO:0046677	01501；
CYBC_BRADU	P51131	fbcH	Cytochrome b/c1	5.919244	0.007996	GO:0016021;GO:0042597;GO:0005886;GO:00	00190;02020

						70469;GO:0009055GO:0020037;GO:0046872;GO:0022904	
ILVC_SYNS3	Q0IC80	ilvC	Ketol-acid reductoisomerase (NADP(+))	4.953258	0.029755	GO:0004455;GO:0000287;GO:0050661;GO:0009097;GO:0009099	00290;00770
TREA_PSEAE	Q9I165	treA	Periplasmic trehalase	3.819662	8.35E-05	GO:0042597;GO:0004555;GO:0015927;GO:0044248;GO:0071474;GO:0005993	00500；
FTHS_DESOH	A8ZZJ0	fhs	Formate--tetrahydrofolate ligase	3.546526	0.006325	GO:0005524;GO:0004329;GO:0035999	00670；
YCJV_ECOL6	Q8FHR3	ycjV	Uncharacterized ABC transporter ATP-binding protein YcjV	3.095866	0.011828	GO:0032991;GO:0140359;GO:0005524;GO:0008643	02010；
CH602_GLUDA	A9HPH6	groEL2	Chaperonin GroEL 2	2.594553	0.038172	GO:0005737;GO:0005524;GO:0140662;GO:0016853;GO:0051082;GO:0042026	03018;
TPIS_LACP7	A9KPF8	tpiA	Triosephosphate isomerase	2.165081	0.008314	GO:0005737;GO:0004807;GO:0006094;GO:0006096	00010;00051
GPMA_POLAQ	A4T096	gpmA	2,3-bisphosphoglycerate-dependent phosphoglycerate mutase	1.968986	0.041331	GO:0046538;GO:0006094;GO:0006096	00010;00680;00260
1433_CANAL	O42766	BMH1	14-3-3 protein homolog	0.131013	0.008704	GO:0009986;GO:0005737;GO:1903561;GO:0005886;GO:0030445;GO:0050815;GO:0001410;GO:0008104;G	04011；

						O:0006109;GO:0007346;GO:0007165	
METK_DESRM	A4J945	metK	S-adenosylmethionine synthase	0.227595	0.006106	GO:0005737;GO:0005524;GO:0000287;GO:0004478;GO:0006730;GO:0006556	00270;00999
ASSY_EHRRW	Q5HBF2	argG	Argininosuccinate synthase	0.244022	0.017353	GO:0005737;GO:0004055;GO:0005524;GO:0006526	00250;00220;00250
PCKA_LACE2	C4Z0Q6	pckA	Phosphoenolpyruvate carboxykinase (ATP)	0.247799	0.048896	GO:0005737;GO:0005524;GO:0004612;GO:0006094	00010;00020;00620
DHE3_BACTN	P94598	gdhA	Glutamate dehydrogenase	0.24843	0.017762	GO:0005829;GO:0004352;GO:0004354;GO:0006537	00910;00250;00220
DHE2_BACFR	P94316	gdhB	NAD-specific glutamate dehydrogenase	0.257453	0.00033	GO:0004352;GO:0006520	00910;00250;00220
RPOC_CLOD6	Q18CF3	rpoC	DNA-directed RNA polymerase subunit beta'	0.261917	0.038998	GO:0000428;GO:0003677;GO:0003899;GO:0000287;GO:0008270;GO:0006351	03020;

Table S4. Differential protein in GP1H group.

ID	Accession	Gene Name	Description	FC	pValue	GO	KEGG
ACDS_CLOAB	P52042	bcd	Acyl-CoA dehydrogenase, short-chain specific	6.52	0.00	GO:0003995;GO:0004085;GO:0050660;GO:0019605	00640;00650;00071;00280;00410
6PGL_STRCO	Q9XAB7	pgl	6-phosphogluconolactonase	6.18	0.00	GO:0005737;GO:0017057;GO:0005975;GO:0009051	00030;
ENO_SO	Q01YD1	eno	Enolase	5.98	0.02	GO:0009986;GO:0005	00010;0

LUE						576;GO:0000015;GO:0000287;GO:0004634;GO:0006096	00680;03018
FTHS_CLOD6	Q189R2	fhs	Formate--tetrahydrofolate ligase	3.41	0.02	GO:0005524;GO:0004329;GO:0035999	00670;00670
CH60_LACP7	A9KSJ1	groEL	Chaperonin GroEL	3.38	0.01	GO:0005737;GO:0005524;GO:0140662;GO:0016853;GO:0051082;GO:0042026	03018;
G3P_STRPQ	P0DB19	gap	Glyceraldehyde-3-phosphate dehydrogenase	3.20	0.04	GO:0005737;GO:0004365;GO:0051287;GO:0050661;GO:0006006;GO:0006096	00010;
TPIS_CLOBA	B2UY22	tpiA	Triosephosphate isomerase	2.79	0.04	GO:0005737;GO:0004807;GO:0006094;GO:0006096	00010;00051;00562
PCTB_PSEAE	Q9HW91	pctB	Methyl-accepting chemotaxis protein PctB	2.72	0.02	GO:0016021;GO:0005886;GO:0016597;GO:0006935;GO:0043200;GO:0007165	02020;02030
ILVC_CLOBA	B2UYT8	ilvC	Ketol-acid reductoisomerase (NADP(+))	2.71	0.01	GO:0004455;GO:0000287;GO:0050661;GO:0009097;GO:0009099	00290;00770
GLPK_CARHZ	Q3AB25	glpK	Glycerol kinase	2.52	0.00	GO:0005524;GO:0004370;GO:0019563;GO:0006071;GO:0006072;GO:0016310	00561;
VATA_ACET2	A3DHP0	atpA	V-type ATP synthase alpha chain	2.46	0.01	GO:0045259;GO:0005524;GO:0046933;GO:0046961;GO:0042777	00190;
TPIS_LACP7	A9KPF8	tpiA	Triosephosphate isomerase	2.27	0.01	GO:0005737;GO:0004807;GO:0006094;GO:0006096	00010;00051
ASSY_EHRRW	Q5HBF2	argG	Argininosuccinate synthase	2.06	0.01	GO:0005737;GO:0004055;GO:0005524;GO:0006526	00250;00220
SUCD_RICFE	Q4ULQ8	sucD	Succinate--CoA ligase [ADP-forming] subunit alpha	1.66	0.02	GO:0000166;GO:0004775;GO:0006099	00020;00640
XYLA_BACFN	Q5LCV9	xylA	Xylose isomerase	0.07	0.01	GO:0005737;GO:0000287;GO:0009045;GO:0042732	00040;00051
SERC_P	A6L9B7	serC	Phosphoserine	0.19	0.03	GO:0005737;GO:0004	00680;0

ARD8			aminotransferase			648;GO:0030170;GO:0006564;GO:0008615	00260;00270;00750
GPMA_POLAQ	A4T096	gpmA	2,3-bisphosphoglycerate-dependent phosphoglycerate mutase	0.27	0.04	GO:0046538;GO:0006094;GO:0006096	00010;00680;00260
DHE3_BACTN	P94598	gdhA	Glutamate dehydrogenase	0.27	0.03	GO:0005829;GO:0004352;GO:0004354;GO:0006537	00910;00250;00220
DHE2_BACFR	P94316	gdhB	NAD-specific glutamate dehydrogenase	0.35	0.01	GO:0004352;GO:0006520	00910;00250;00220
PNP_CYTH3	Q11U61	pnp	Polyribonucleotide nucleotidyltransferase	0.36	0.00	GO:0005737;GO:0000287;GO:0004654;GO:0003723;GO:0006402;GO:0006396	03018;
ENO_BACCZ	Q631M2	eno	Enolase	0.38	0.04	GO:0009986;GO:0005576;GO:0000015;GO:0000287;GO:0004634;GO:0006096	00010;00680;03018
PNP_PARD8	A6LFK9	pnp	Polyribonucleotide nucleotidyltransferase	0.39	0.01	GO:0005737;GO:0000287;GO:0004654;GO:0003723;GO:0006402;GO:0006396	03018;
MDH_BACFN	Q5L8Z8	mdh	Malate dehydrogenase	0.41	0.01	GO:0030060;GO:0019752;GO:0006099	00020;00620;00630;00680;00270
1433_CANAL	O42766	BMH1	14-3-3 protein homolog	0.41	0.05	GO:0009986;GO:0005737;GO:1903561;GO:0005886;GO:0030445;GO:0050815;GO:0001410;GO:0030447;GO:0044182;GO:0036180;GO:0008104;GO:0006109;GO:0007346;GO:0007165	04011;
SECDF_THET8	Q5SKE6	secDF	Protein translocase subunit SecDF	0.42	0.02	GO:0016021; GO:0005886; GO:0015450; GO:0065002;GO:0006	03060;03070;02024

UXAC_PHOV8	A6L4U4	uxaC	Uronate isomerase	0.42	0.05	605;GO:0043952 GO: 0008880; GO:0006064	00040;

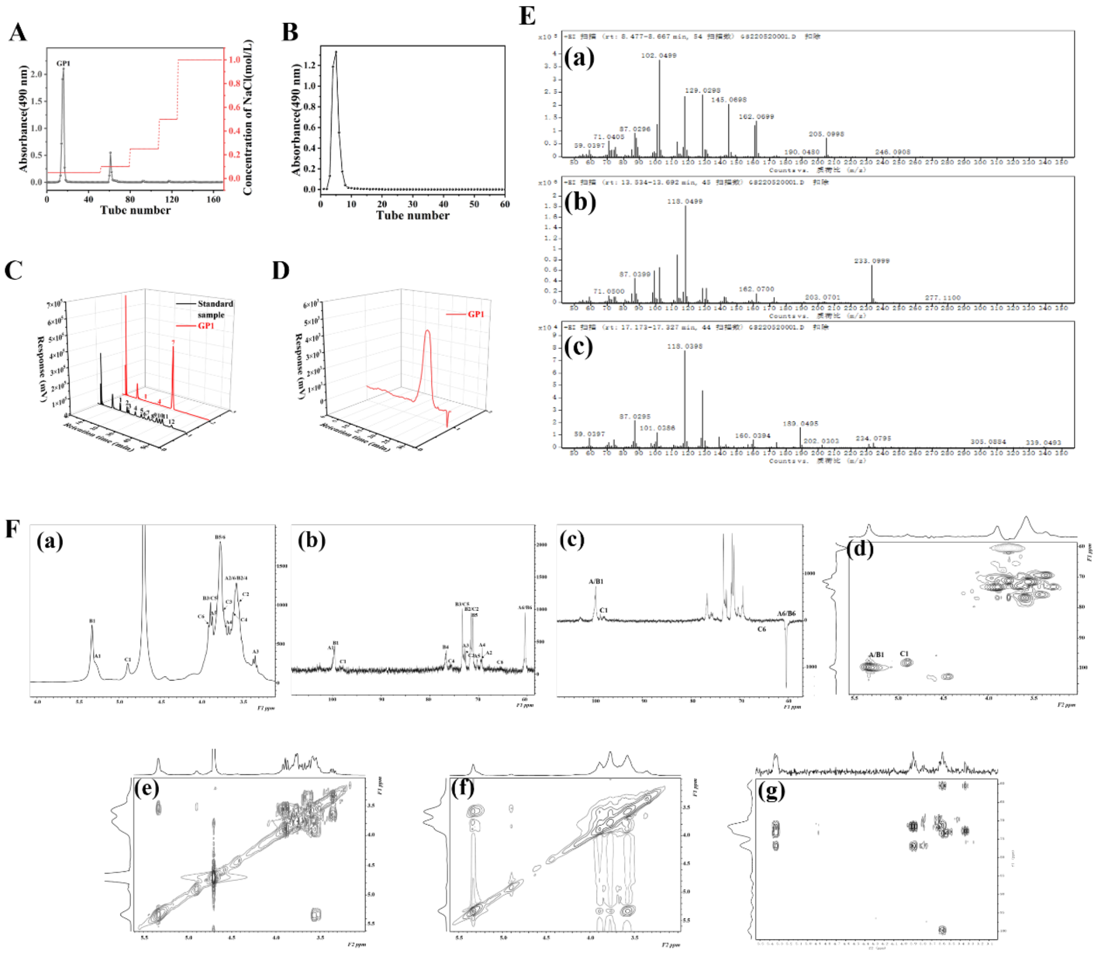

Figure S1. Purification, basic properties and glycoside bond analysis of GP1. (A) Elution curve of GP1 by DEAE-52 cellulose anion-exchange column chromatography. (B) Elution curve of Sephadex G-100 column chromatography of GP1. (C) Monosaccharide composition of GP1 and Standard sample (1-Man, 2-Rib, 3-Rha, 4-GlcA, 5-GalA, 6-GlcNAc, 7-Glc, GalNAc, 9-Gal, 10-Xyl, 11-Ara, 12-Fuc). (D) The gel filtration chromatograms of GP1. (E) Ion peak of sugar residue fragment of GP1: (a) α-D-Glc*p*-(1→; (b) →4)-α-D-Glcp-(1→; (c) →4,6)-α-D-Glcp-(1→. (F) NMR spectra of GP1. (a) ^{1}H-NMR; (b) ^{13}C-NMR; (c) Dept 135 NMR; (d) HSQC; (e) ^{1}H-^{1}H COSY; (f) NOESY; (g) HMBC. (The article on structural analysis of GP1 is being published.)

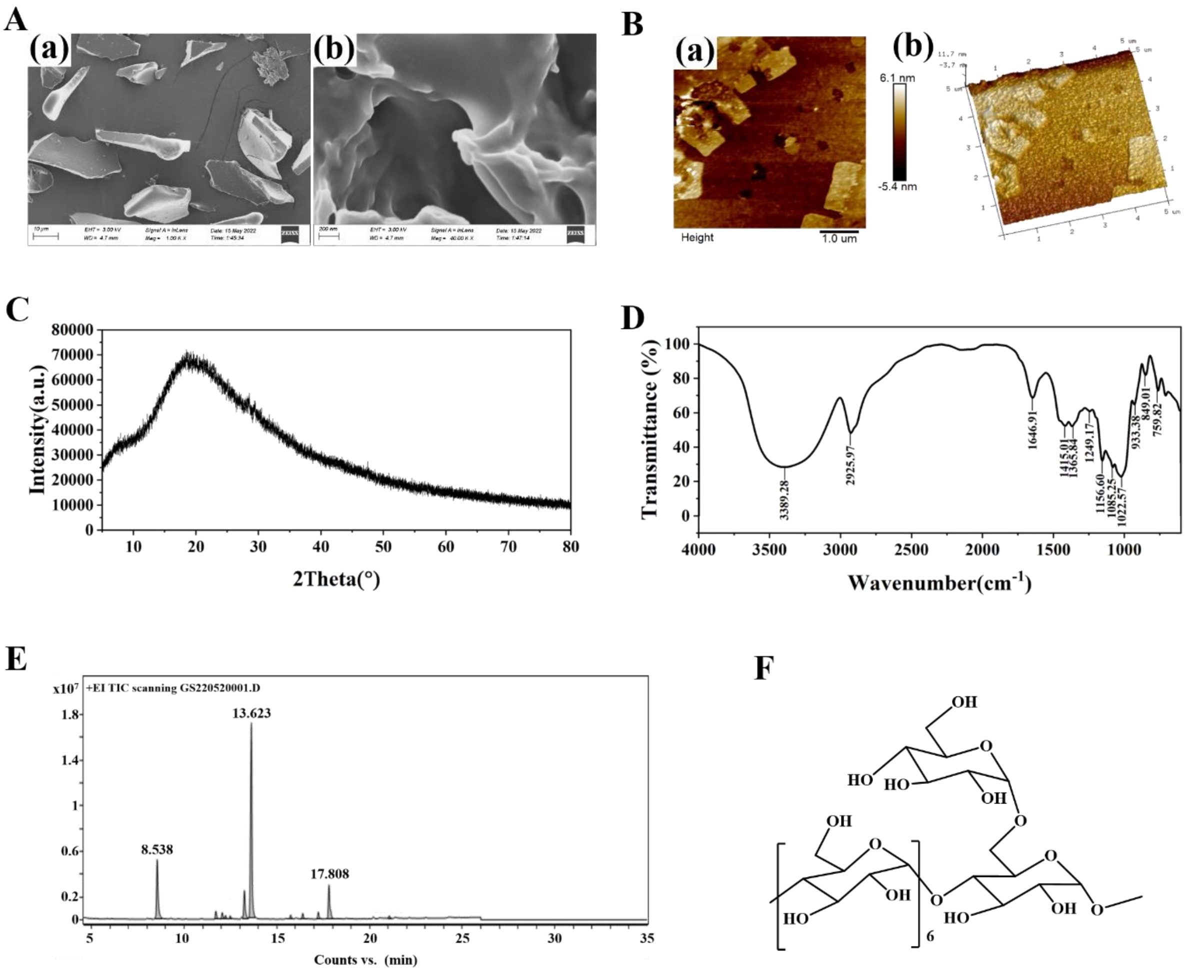

Figure S2. Structural analysis of GP1. (A) SEM ((a) 1000 x ;(b) 40000 x). (B) AFM ((a) 2D; (b) 3D); (C) XRD; (D) FT-IR; (E) TIC profile of GP1; (F) Presumed structure of GP1. (The article on structural analysis of GP1 is being published.)

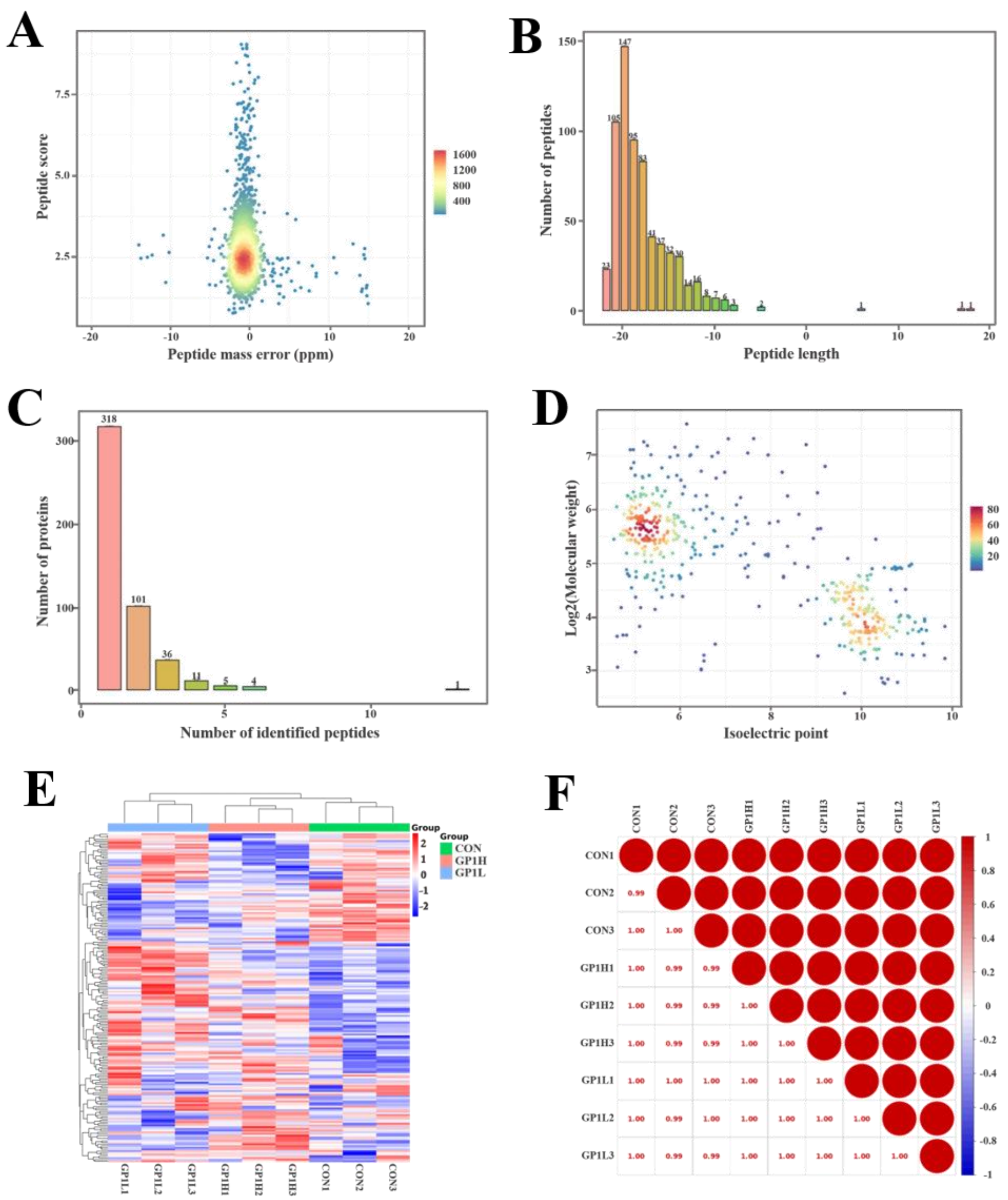

Figure S3. Detection and analysis of differential proteins in gut microbiota. (A) Overall deviation statistical chart. (B) Statistical diagram of peptide length. (C) Statistical chart of the number of peptide segments matched by protein. (D) Identification of isoelectric point and molecular weight scatter plot of protein. (E) Global analysis clustering heat map. (F) Sample correlation matrix diagram.

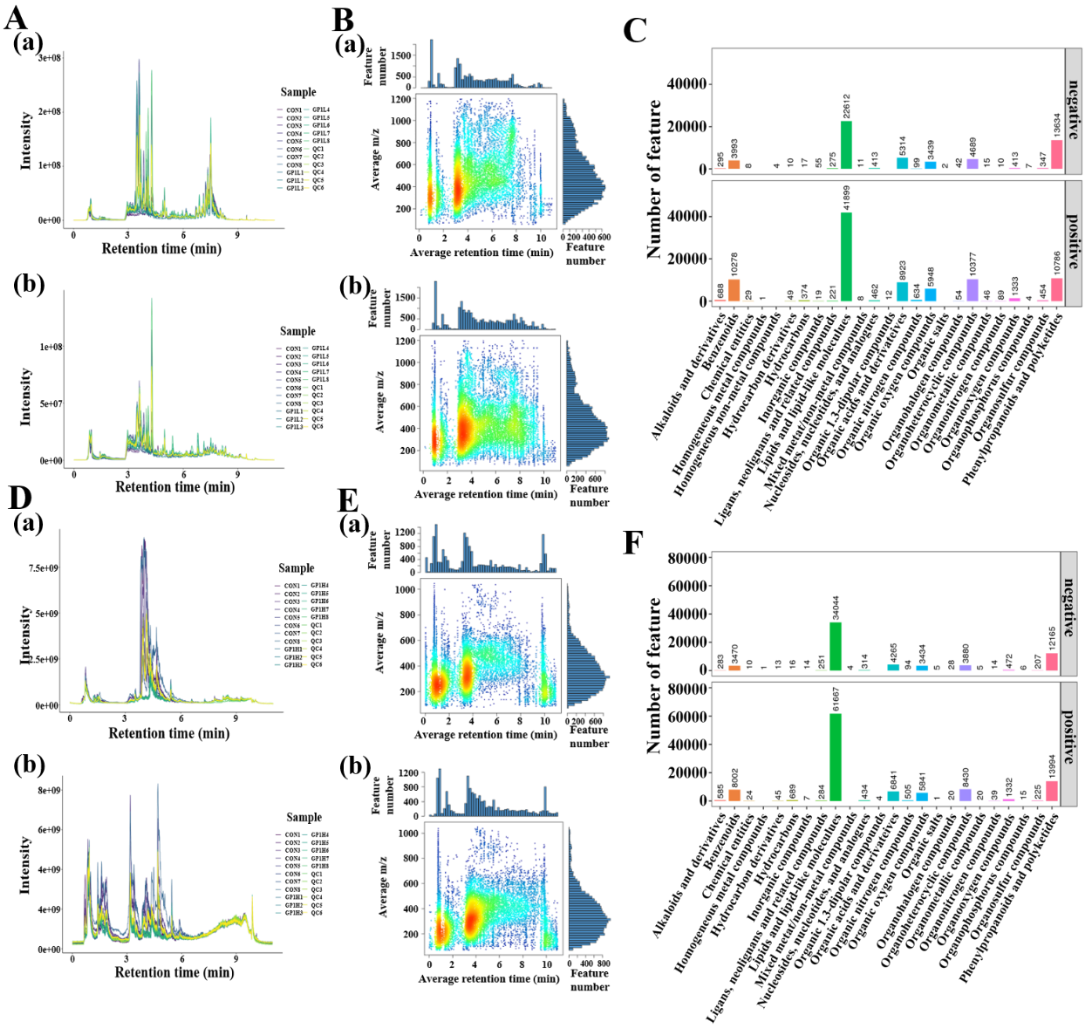

Figure S4. Detection and identification of metabolites in the fecal treated with GP1 and saline. (A) Total Ion Chromatogram – GP1L; (a) pos; (b) neg. (B) Metabolites m/z-rt distribution – GP1L: (a) pos; (b) neg. (C) Classification of metabolites – GP1L. (D) Total Ion Chromatogram – GP1L; (a) pos; (b) neg. (E) Metabolites m/z-rt distribution – GP1L: (a) pos; (b) neg. (F) Classification of metabolites – GP1L.
